# Influence Analysis of Education Policy on Migrant Children’s Education Integration Using Artificial Intelligence and Deep Learning

**DOI:** 10.3389/fpsyg.2022.910692

**Published:** 2022-06-17

**Authors:** Zhen Chen, Zhitian Song, Sihan Yuan, Wei Chen

**Affiliations:** ^1^School of Marxism, Northeastern University, Shenyang, China; ^2^The Experimental High School Attached to Beijing Normal University, Beijing, China; ^3^School of Tunburi University in Thailand, Bangkok, Thailand; ^4^Zhongnan University of Economics and Law, Huazhong Agricultural University, Wuhan, China

**Keywords:** artificial intelligence, deep learning, education policy, effect evaluation model, migrant children

## Abstract

This work intends to solve the problem that the traditional education system cannot reasonably adjust the educational integration of children with the arrival of labor force in a short time, and support the education of migrant children (MC) in the education policy (EP) to integrate them into the local educational environment as soon as possible. Firstly, this work defines the surplus labor force and MC. Secondly, the principles of Artificial Intelligence (AI) and Deep Learning (DL) are introduced. Thirdly, it analyzes the education of MC and relevant policies, and the data of the education effect of MC are collected and the evaluation effect model is built. Finally, the evaluation model of MC’s education effect is applied to test the effect of EP. The results show that using AI technology combined with DL technology to model the education effect of MC can establish an effective and accurate evaluation model of the education effect of MC, effectively evaluate the impact of local education policies on the education of MC, and give an effective effect analysis of relevant education policies in each period. The result of Adaptive Resonance Theory (ART)–Back Propagation algorithm is 65 ∼ 96%, which is much higher than the efficiency of traditional algorithms. This shows that the education integration evaluation model of MC based on AI technology and DL technology can effectively and accurately evaluate the integration effect of MC on the local education system, and then provide reference for local and even national adjustment of education policies. The results provide a new idea for the application of new technology in EP.

## Introduction

With the continuous development of social economy, all walks of life in China have developed rapidly, among which the construction industry has created a large number of jobs. The surplus rural labor force was liberated from the land, entered the vast economically developed areas and cities, and became an excellent labor force. With the transfer of rural labor force to cities and towns, there are a large number of migrant children (MC), which has put some pressure on the construction of local infrastructure and the balance of education system, and further affected the education level of MC (Tuangratananon et al., 2019). Because MC may become human resources for local urban development in the future, the education level of MC is closely related to the future urban development and the stable development of national society. The core technologies of Artificial Intelligence (AI) mainly include: Deep Learning (DL), Computer Vision, Natural Language Processing, and Data Mining. The core idea of AI technology is to construct an intelligent artificial system, use machines to imitate human beings to complete a series of actions, and realize advanced behaviors such as understanding, thinking, reasoning, and problem solving. Therefore, the work plans to apply AI technology to the analysis of education policy (EP), thus analyzing the education problems of MC and further solve the impact of EP on local education level.

Deep Learning is based on big data, and its implementation path is cloud computing. As long as there is sufficient data and fast enough computing power, the results will be more accurate. Machine learning is an important method to realize AI. It is to use algorithms to analyze data, learn from it and automatically summarize it into a model, and finally use the model to make inference or prediction. Zhang et al. (2021a) proposed a multi-source sparse attention convolution neural network model to predict students’ course grades. The model is used to collect and process students’ grades, and the relationship between courses is found through global attention strategies, which is integrated into multi-source features. The experimental results showed that the designed model could explain students’ performance through the generated curriculum relationship. Zhang et al. (2021b) studied the impact of machine learning and data mining on students’ performance, processed and analyzed the data in the dataset through five processes: data collection, problem formalization, model design, data prediction, and application effect, and finally summarized the discussion on the shortcomings of current teaching work. Zhang et al. (2020) proposed a student knowledge diagnosis model to diagnose students’ learning status by learning meta knowledge dictionary from students’ answers. The results indicated that the designed method provided effective student knowledge diagnosis and had better performance than similar models (Zhang et al., 2020). Therefore, in the current research, the application of DL technology to education needs to be further improved, thereby choosing more appropriate technology to promote the development of education.

Under the rapid development of AI technology, this work uses back propagation neural network (BPNN) technology to construct the evaluation model of the effect of EP on the educational integration of MC. Firstly, it analyzes the scope of MC caused by migrant surplus labor force. Secondly, it introduces AI and DL technology, and analyzes the supporting education policies of non-registered permanent residents. Thirdly, the data of policy implementation effect are collected and the evaluation model of MC’s education effect is established. Finally, the effect evaluation model of MC’s education is applied to evaluate and modify the effect of EP. The research provides a new application perspective for computer technology to promote the improvement of educational work level.

## Education Policy Evaluation Modeling

### Migrant Children, School Enrolment, Education Policy, and Social Integration

(1) MC

Migrant children groups refer to the transient population of China’s children aged 0–17. Floating population refers to residents who have left their place of domicile for more than half a year, which has increased since the Reform and Opening Up policy in the 1980s (Toker, 2021; Miller et al., 2022).

After the Reform and Opening Up policy, with the influx of a large number of the rural labor force to cities and towns, the education of MC has gradually become the focus and difficulty of the Compulsory Education (CE) system in China. In 2014, school-enrolled MC in cities reached 12.947 million, 79.5% in public schools. In 2015, nine provinces in China had 26.72 million left-behind children (LBC) in rural areas, accounting for 66% of the total. The rural LBC in some provinces accounts for up to 40% of the local rural child population, such as Chongqing, Sichuan, and Hubei. From 2000 to 2015, the scale of MC and LBC increased significantly. However, the rural LBC decreased in 2015 compared with 2010, while the scale of MC increased, which is consistent with the general trend of changes in urban and rural population structure in the process of urbanization. *China Human Development Report 2016* points out that the education of MC is far from optimistic. The central government promises to ensure that MC can receive CE on time in the access area (Liang et al., 2020). However, school-age children still face some difficulties entering school, especially in public schools requiring a lot of written confirmation. In addition, some local governments responsible for raising funds are inadequate to support a mass of MC.

Tables 1, 2 list the promulgation of the Eps for MC and the schooling issues of MC, respectively.

**TABLE 1:** Promulgation of the education policys (Eps) for migrant children (MC).

Time	Policy name	Policy interpretation
1998	The *Ministry of Education* and *Public Security* jointly issued the “Interim Measures for MC and Adolescents to Attend School.”	The policy aims to solve the problem of MC enrollment mainly through encouraging full-time public primary and secondary schools in relocated areas to receive transferred students and authorizing schools to charge the temporary schooling fee.
1998	“Notice on Opinions on Solving Several Prominent Problems in Current Household Registration Management”	It aims to control the population of big cities strictly.
2001	The decision of the *State Council* on the reform and development of basic education	Aiming at the enrollment of school-age MC, the policy focuses on the management of local governments and full-time public primary and secondary schools and adopts various forms to protect the right of the Department of civil affairs to obtain CE according to law.
2003	The *State Council* promulgated the “Opinions on Further Improving the Compulsory Education of Migrant Children.”	It further clarified the policy orientation of paying attention to the relocated areas and public schools.
2006	Compulsory Education Law	It clarified the right of MC to education again in the form of law.
2015	Notice of the *State Council* on Further Improving the Funding Guarantee Mechanism for Urban and Rural Compulsory Education	MC enjoying the policies of Two Exemptions and One Subsidy and Per-Student Operational Expenditure of Education can continue to enjoy this right in the relocation place, which has brought certain progress.

*Two exemptions and one subsidy indicates that China implements the policy measures of free miscellaneous fees, free book fees and subsidizing the living expenses of boarding students in the stage of compulsory education.*

**TABLE 2:** School enrollment of migrant children (MC) in the past.

Requisite formalities	Specific explanation
Residence permit	Outsiders who rent houses need to pay taxes through the landlord’s real estate card and ID card.
The certifying document concerning family planning	Linking enrollment with family planning
Certifying document of no guardianship conditions at the outflow place	
Certificate of continuous social security payment	Persons whose Social Insurance is interrupted once or who have insufficient contributions to Endowment Insurance, Medical Insurance, Unemployment Insurance, Work-Related Injury Insurance, or Maternity Insurance cannot apply for it.
Proof of employment	Some areas require the child’s parents to work in the same place of residence to arrange formal enrollment in the destination area.

(2) Enrollment rate

CE has been popularized throughout China, basically achieving the goal of universal schooling and eliminating gender inequality. Meanwhile, with the development of the social economy, the gap between urban and rural areas, especially the gap between facilities, has narrowed rapidly. Nevertheless, most high-quality talents and teachers are unwilling to work and live in rural, underdeveloped areas. As a result, the gap in the CE quality between urban and rural areas may become even more prominent. Due to specific historical reasons, such unbalanced development and resource allocation also generally exist in the same urban area. Therefore, the “school choice” (paying to enroll one’s children in better schools) phenomenon has become a severe issue in China for a long time. A study shows significant differences in the quality of schooling of children of local registered residents, migrant workers, and low-income families. Pre-school and high school education are not yet universal in China. There are significant regional differences in the opportunities for urban and rural children to receive pre-school education and high school education. Table 3 shows the changes in the gross enrollment rate of CE in China (Chen et al., 2019).

**TABLE 3:** Changes in China’s gross enrollment rate.

Year	1949	1965	1978	1990	2000	2010	2012	2015	2016	2017	2018	2019
Gross enrolment ratio (%)	1.69 _	21.8	67.7	68.1	90.6	96.5	99.7	99.9	100	100	100	100

(3) EP

Education policy is incorporated into the national public policy, which is crucial for the development of national education. The fairness of national education also reflects the fairness of education in the country. The EP proposals aim to enable every school-age child to receive education equally, enjoy the same educational resources, and receive the same educational opportunities. This work defines the EP as the policies and regulations determined by national legislation to ensure that MC in the stage of CE can obtain the same educational opportunities as local children. Its purpose is to ensure the education of MC, especially to promote their integration into local society (Hu and Wu, 2020).

The deficiency and weakness of EPs are the main reasons for the imbalance.

Education policy shortfalls mean no corresponding ordinances to regulate and guide the affairs or activities in a specific time and space. Most Chinese EPs seem to be passively activated. In other words, no active or targeted policy will be proposed before the education problem becomes serious. On the one hand, the evolution of the problem usually goes through a cycle from latent to obvious, from mild to severe. On the other hand, a new policy needs some time to be implemented. Thus, there is often a time lag from promulgation to implementation and from implementation to effect.

The so-called weak EPs indicate that the current EP cannot achieve the expected effect, nor can it regulate and manage such practices, involving two aspects. First of all, the role of the policy itself is limited due to the particularity of the problem, government capacity, policy programs, policy tools, the acceptance of target groups, and other reasons. Secondly, there are subjective errors in the formulation and implementation of EPs.

(4) Social integration

The social integration ability assessment tool can help children’s individualized education planning team make decisions and set priorities to meet children’s needs for education. The evaluation of social integration ability covers subjects’ negative behavior, collective ability, social ability, and independent learning ability.

Migrant children’s social integration refers to the process of communication, adaptation, and acceptance between MC and various social groups in the immigration area. It aims to eliminate all adverse differences between MC and local children and help them connect with local life (Ge, 2019; He et al., 2021).

### Artificial Intelligence Technology

Artificial Intelligence is a theory, method, technology, and application system using digital computers or machines controlled by digital computers to simulate, extend, and expand human intelligence, perceive the environment, acquire knowledge, and use knowledge to obtain the best results. The core idea of AI is to construct intellectual systems. The core technologies of AI mainly include DL, Computer Vision, Natural Language Processing, and Data Mining (Ting et al., 2019; Ryuichi et al., 2021). Unlike traditional programming languages and development software, AI uses large amounts of data to feed ML, a process called training. BPNN is a concept proposed by scientists led by Rumelhart et al. (1986). It is a multi-layer feedforward neural network trained according to the Error BPNN algorithm. It has become the most widely used Neural Network. AI is a knowledge project that uses machines to imitate human beings to complete a series of actions to achieve functions similar to human understanding, thinking, reasoning, and problem-solving.

(1) DL

Deep Learning was first introduced as an extension of ML in 2006 and has seen extensive applications ever since. In 2012, a research team at Stanford University built a training model using a parallel computing platform with 16,000 CPU cores named the Deep Neural Network (DNN). The DNN has made a tremendous breakthrough in speech and image recognition. Later, in 2016, AlphaGo (an artificial go software) developed *via* DL beat Li Shishi, the world’s top go master. This event marked the beginning of a DL-based new technological revolution involving almost all high-tech companies worldwide, with which numerous research institutes and Research and Development teams were established.

Machine Learning (ML) technology studies how computers simulate or implement animals’ learning behavior to learn new knowledge or skills, update existing data structures and enhance program performance. Statistically, it predicts data distribution, learns a model from the data features, and makes predictions. Such operations typically require identically distributed test data and training data. Its essential feature is to imitate transmitting and processing information between brain neurons. ML has seen its most significant applications in Computer Vision and Nature Language Processing. Obviously, DL strongly promotes the development of Artificial Neural Networks (ANNs) in ML, and ANN is also the main implementation path of DL. Simply put, DL is a somewhat improved ANN algorithm to simulate human neurons. Each neuron receives information and transmits the processed data to all adjacent neurons (Bossuyt et al., 2020).

(2) Artificial neuron

**FIGURE 1:**
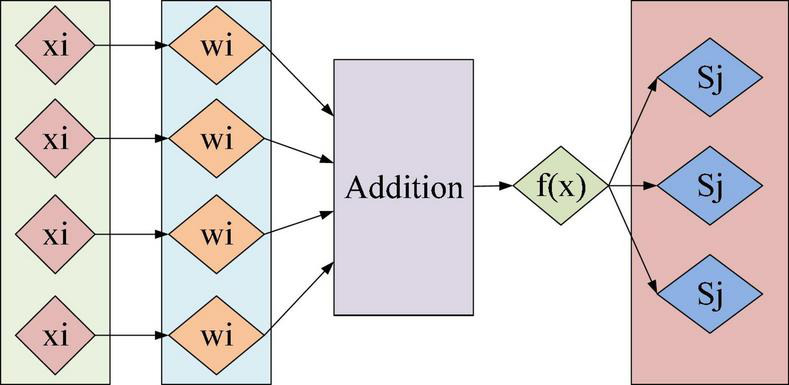
Schematic principle of artificial neuron.

An artificial neuron is a mathematical model imitating the primary operation function of biological neurons and inheriting some operation effects of biological neurons. The artificial neuron receives the signal from the front neuron and weights each given signal. This neuron will exhibit a corresponding activation state under all weight states’ joint action. Figure 1 reveals the rationale.

Eq. (1) describes the artificial neuron.


(1) $$f\left(x\right)=\sum_{i=1}^{n}x_{i}w_{i}$$

In Eq. (1), *f*(*x*), *x*_*i*_, and *w*_*i*_ represent the final output state, the input signal, and the input signal weight, respectively. There are *n* groups in total.

A neuron will output a specific value with a given input signal. Each neuron corresponds to a threshold. If the input sum of the neuron is greater than its threshold, the neuron gets into an active state; otherwise, it will show an inhibitory state. The transfer functions of artificial neurons are as follows (Granados et al., 2021).

a. The linear function is expressed as Eq. (2).


(2) f(x)=kx

b. Eq. (3) indicates the slope function.


(3) $$\begin{array}{c} f\left(x\right)=\alpha \left(x\geq \theta \right)\\ f\left(x\right)=kx\left(-\theta <x<\theta \right)\\ f\left(x\right)=-\alpha \left(x\leq \theta \right) \end{array}$$

Then, Eq. (4) signifies the transition function.


(4) $$\begin{array}{c} f\left(x\right)=\alpha \left(x\geq \theta \right)\\ f\left(x\right)=\beta \left(x\leq \theta \right) \end{array}$$

Eq. (5) expresses the Sigmoid function:


(5) $$f\left(x\right)=a+\frac{b}{1+\exp\left(-dx\right)}$$

The choice of a transfer function should follow a specific application range. The linear function will amplify the output signal, and the nonlinear slope function can neutralize network performance degradation. The hidden layer reported here uses the S-type function. In Eq. (3) ∼ Eq. (5), parameters α, β, *d*, *a*, *b*, and θ are fixed, and *x* and *f*(*x*) represent the input and output, respectively (Nicolas et al., 2019).

(3) BPNN neuron

Like artificial neurons, BPNN neurons also use weight and summation. This work denotes *x*_*i*_ as the input of the neuron *i* and *w*_*ji*_ as the weight of the connection between the neuron *i* and the neuron *j*. Besides, *b*_*j*_ represents the threshold, *y*_*j*_ stands for the output of the neuron *j*, and *f*(*x*) reveals the transfer function (Srivastava and Ahmed, 2021).

The net output of the neuron *j* is presented in Eq. (6). When threshold = 0, *b*_*j*_ = 0.


(6) $$S_{j}=\sum_{i=1}^{n}w_{ji}\cdot x_{i}+b_{j}$$

**FIGURE 2:**
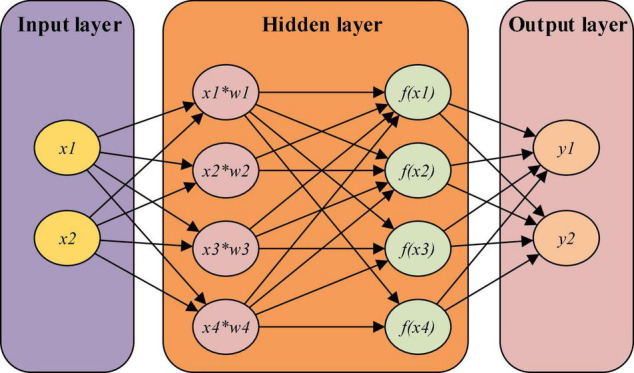
Back propagation neural network (BPNN) structure.

Back propagation neural network is a multi-layer feedforward Neural Network characterized by signal forward transmission and error backward propagation, as depicted in Figure 2. Forward transmission processes the input signal layer by layer from the input layer through the hidden layer to the output layer. Each layer’s neuron state only affects the next-layer neuron state. Error backpropagation will be activated when the output deviates too much from the expected output to adjust the network weight and threshold according to the prediction error. In this way, the predicted output of BPNN approximates the expected output infinitely until the iteration is completed (Ruff et al., 2021). The algorithm flow of BPNN is first network training and then prediction. Training helps the network develops associative memory and prediction ability. The training steps include network initialization, hidden layer output calculation, output layer output calculation, input layer output calculation, error calculation, weight update, and checking whether the algorithm iteration is completed. If not, restart from the second step.

The basic idea of the BPNN algorithm is to split the learning process into signal forward propagation and error backpropagation.

During forward propagation, the input samples are transmitted from the input layer, processed layer by layer by hidden layer, and then transferred to the output layer. If the actual output layer output is inconsistent with the expected output (teacher signal), the network will turn to the error backpropagation stage.

The output is transmitted back to the input layer by layer through the hidden layer in some form during backpropagation. The error is allocated to all units of each layer to obtain the error signal of each layer unit. This error signal can adjust each unit’s weight to complete the Neural Network memory operation process.

Eqs (7, 8) express the propagation process, in which Eq. (7) represents the output of the hidden layer node, and Eq. (8) is the output of the output layer node.


(7) $$z_{k}=f_{1}\left(\sum_{i=0}^{n}v_{ki}x_{i}\right),\left(k=1,2,\cdots ,q\right)$$

(8) $$y_{j}=f_{2}\left(\sum_{i=0}^{n}w_{jk}z_{k}\right),\left(j=1,2,\cdots ,m\right)$$

In Eqs (7, 8), *n*, *q*, and *m* represent the number of nodes in the input, hidden, and output layers; *v*_*ki*_ denotes the weight of the connection between the input layer and the hidden layer; *w*_*jk*_ stands for the weight of the connection between the output layer and the hidden layer; *f*_1_(*x*) indicates the transfer function of the hidden layer; *f*_2_(*x*) denotes the transfer function of the output layer.

### Text Extraction Algorithm Based on Back Propagation Neural Network

**FIGURE 3:**
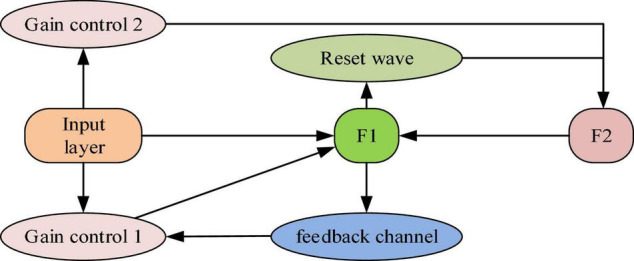
Structure of the ART-BP algorithm.

Policy documents related to EP and MC are collected in the research. The processing algorithm is designed according to the different criteria in the documents, such as government capacity, policy options, policy tools, acceptance of target groups, as well as negative behaviors of social integration capacity, collective ability, and social ability, independent learning ability. Besides, the Adaptive Resonance Theory (ART) is introduced into the BPNN algorithm to adapt to text information extraction, forming the ART-BPNN algorithm. The model consists of two subsystems to analyze and process various events: the attention subsystem and the adjustment subsystem (Veckot and Gupta, 2020; Ren et al., 2022). Figure 3 shows the structure of the ART-BP algorithm.

In Figure 3, F1 and F2 represent the Neural Network’s hidden and output layers, respectively (Bassam et al., 2020; Rai and Sakthivel, 2020). The adjustment subsystem contains a reset wave channel. Eq. (9) indicates the weight coefficient *W*_*ij*_.


(9) $$\frac{dW_{ij}}{dt}=K_{1}f\left(x_{i}\right)\left[-W_{ij}E_{ij}+h\left(x_{j}\right)\right]$$

In Eq. (9), *f*(*x*_*i*_)represents the output after neuron *i* is transformed; *h*(*X*_*i*_) denotes the output after neuron *j* is transformed; *E*_*ij*_ and *K*_1_ are the relevant parameters. Eq. (10) ∼ Eq. (12) express these variables and functions.


(10) $$E_{ij}=h\left(X_{i}\right)+L^{-1}\sum_{k\neq i}h\left(X_{k}\right)$$

(11) $$K_{1}=KL$$

(12) $$\frac{dW_{ij}}{dt}=Kf\left(x_{i}\right)\left[\left(1-W_{ij}\right)Lh\left(X_{i}\right)-W_{ij}\sum_{k\neq i}h\left(x_{j}\right)\right]$$

In Eq. (10) ∼ Eq. (12), *L*^−1^ signifies the parameter setting. Eq, (11) shows the relationship between *K*_1_ and *L*. When the output of neuron *j* in layer F is positive, neuron *i* in layer F can affect the change of weight coefficient.

### Experimental Parameter Setting

**FIGURE 4:**
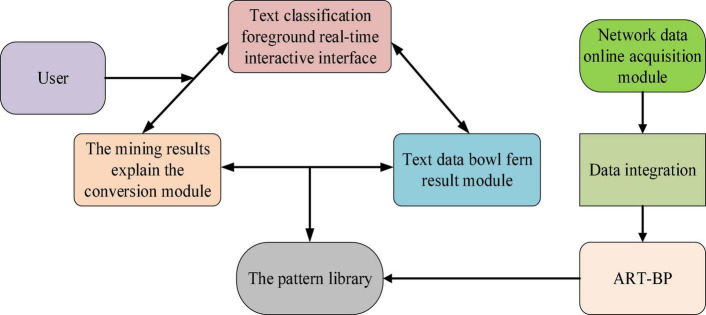
Structure of text classification system based on ART-BP model.

The final comprehensive evaluation of fusion effect needs to use a single input neuron. In addition, the implementation evaluation of EP also needs two output neurons for output. Therefore, the output layer of the basic network structure needs three neurons (Yao and Wang, 2019; Veluri et al., 2022). The previous output parameters need 60 neurons corresponding to the input layer, while the output layer needs 3 neurons. For hidden layer neurons, generally only one hidden feedforward neural network is needed to realize the approximation of the application function in all cases (Wodecki, 2019). The structure and weight coefficients of BPNN need to be determined by learning method to form a correct mapping. The learning process of neural network is to solve a set of weight coefficients that can make the error function reach the minimum value under a certain network structure (Bhattacharya et al., 2022). Figure 4 shows the structure of text classification system of ART-BP model.

Eq. (13) ∼ Eq. (15) indicate the BPNN algorithm’s global error. The Mean Square Error in the training process is calculated by Eq. (14). Eq. (15) describes the operation accuracy of the algorithm.


(13) $$E_{p}=\frac{1}{2}\sum_{j=1}^{m}{\left(t_{j}^{P}-y_{j}^{P}\right)}^{2}=\frac{1}{2}\sum_{p=1}^{P}\sum_{j=1}^{m}{\left(t_{j}^{P}-y_{j}^{P}\right)}^{2}$$

(14) $$MSB=\frac{1}{mp}\sum_{p=1}^{P}\sum_{j=1}^{m}{\left(\bar{y_{kj}}-y_{kj}\right)}^{2}$$

(15) $$P\left(T\right)=\frac{\sum_{c}A_{1}\left(c,T\right)}{\sum_{c}A_{1}\left(c,T\right)+A_{2}\left(c,T\right)}$$

In the above three equations, *E*_*p*_ represents the error; $t_{j}^{P}$ is the actual output of the *j*-th time; $y_{j}^{P}$ denotes the expected output of the *j*-th time; *m* and *P* indicate the number of output nodes and the number of training samples; *y*_*kj*_ signifies the actual output of BPNN; $\bar{y_{kj}}$ stands for the expected output of BPNN; *A*_1_(*c*, *T*) and *A*_2_(*c*, *T*) mean the number of correctly classified documents and incorrectly classified ones; *c* and *T* are the data category and object.

## Performance Test of the Evaluation Model

### Education Policy for Migrant Children

**FIGURE 5:**
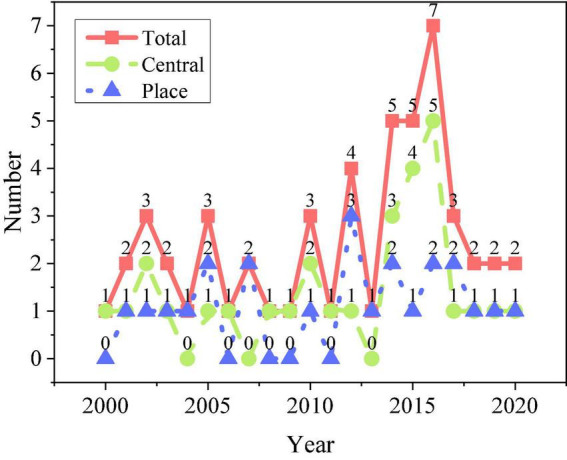
Number of documents issued on education policys (EPs) for migrant children (MC).

The research dataset is the relevant documents about EP of MC from 2010 to 2020, and the original documents are processed by text extraction algorithm. The statistical results of the number of documents issued by the collected policy documents related to the education of MC are shown in Figure 5.

Figure 5 showed that China issued a total of 35 relevant policy documents on the education of MC from 2010 to 2020, accounting for 67% of all the documents issued since the founding of the People’s Republic of China. Between 2014 and 2016, the number of policy documents issued grew the fastest, a total of 17 copies, the same number of publications as in the 15 years from 1996 to 2010. This phenomenon is because the central government issued the education development planning document for the next 10 years in 2010. It pointed out the need to establish the fairness, universality, and public welfare of the national CE stage, build a basic public education system covering urban and rural areas and focus on protecting the educational rights of MC and promoting the integration of education.

**FIGURE 6:**
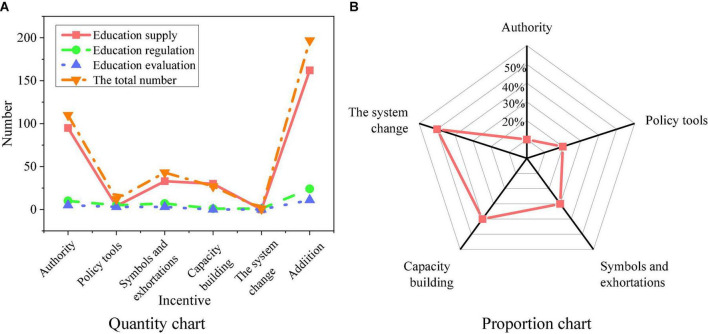
Use of education policy (EP) tools for migrant children (MC). **(A)** Quantity chart of different policy tools; **(B)** proportion chart of different policy tools.

The content of policy texts is counted, aiming at the usage of EP tools for MC. Figure 6 provides the statistical analysis results.

The policy tools in Figure 6 include five means: authority, incentive means, symbol and exhortation, capacity building, and system change. The use of authority reaches 55.9%, accounting for the largest proportion; the most frequently used tool is capacity building, reaching 22.8%; the third is the incentive means, reaching 12.7%; the fourth is the symbol and exhortation, reaching 8.1%; the application of system change reaches 0.5%.

### Comparison of Experimental Effects

**FIGURE 7:**
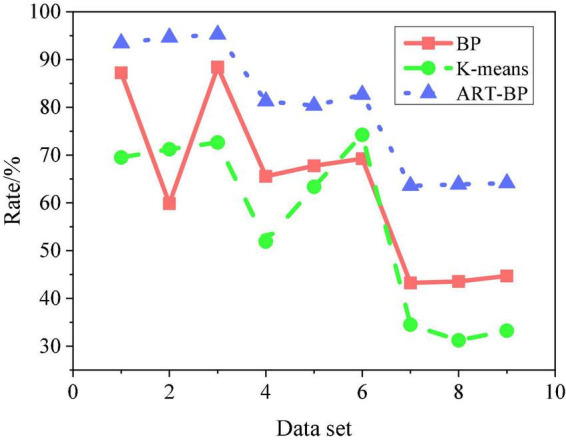
Comparison of experimental results and accuracy of three algorithms.

To obtain accurate experimental results, the output results of the model need to be improved continuously in the experiment. Finally, 67 hidden layer nodes are determined, and the output results of the model are optimized by continuously adjusting the model parameters (Jarbou et al., 2022). The basic data set used here is obtained. In the process of training the model, the text in the data set is divided into training set and test set according to the ratio of 6:3, and further subdivided into 9 text datasets, labeled 1 ∼ 9 respectively. In order to verify the progressiveness of the design algorithm, the experimental results are compared with the traditional BP algorithm and K-means clustering algorithm. The input parameters of the model are the same as those of ART-BP, and the parameters of the model are the optimized parameter settings in similar studies. Finally, the algorithm is tested with 9 marked text datasets. Figure 7 shows the accuracy comparison of the experimental results of the three algorithms.

It can be seen from Figure 7 that the accuracy of the ART-BP algorithm with the 9 data sets are 65∼96%, the results of the BP algorithm are 43 ∼87%, and the results of the K-means algorithm are in the range of 31 % to 75%. The results indicate that the ART-BP algorithm has higher accuracy than the other algorithms. In addition, it performs more stably on different data sets than them. Therefore, the effect of the ART-BP algorithm proposed here is the best, which is much higher than the efficiency of the traditional BP algorithm and the K-means algorithm.

**FIGURE 8:**
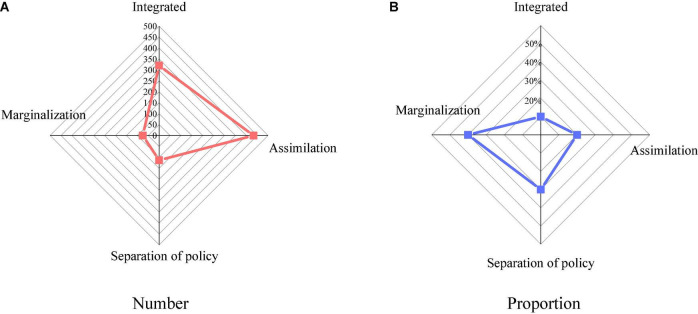
Migrant childrens (MC’s) ways of cultural adaptation. **(A)** Number of Migrant childrens (MC’s) ways of cultural adaptation; **(B)** proportion of Migrant childrens (MC’s) ways of cultural adaptation.

Figure 8 shows the cultural adaptation methods of MC and statistics on the results of strategies and means.

Figure 8 suggests that the most frequently used strategies for MC are the assimilation strategy, followed by the integration strategy, followed by the separation strategy, and the marginalization strategy. In terms of proportion, 47% of MC adopted the assimilation strategy, 35% adopted the integration strategy, 13% adopted the separation strategy, and 9% adopted the marginalization strategy.

## Discussion

According to the research data, there were 16 pieces of EP documents issued between 2016 and 2020. A total of 56 documents came from 17 different ministerial level state organs, with an average of 3 documents issued by each ministry. The State Council issued the most among them, reaching 26, indicating that the state attaches importance to the education of MC. The analysis of policy tools shows that there are relatively few capacity-building tools and system change tools, which reserves space for adjustment and repair for the introduction of follow-up policies and is conducive to the long-term benefits of policy development (Nicolas et al., 2019; Utaminingrum et al., 2021). Huang (2021) studied how AI courses cultivate students’ key abilities, and analyzed the influence of AI education on students’ knowledge ability, team ability, and learning ability in the stage of basic education. Additionally, they identified the problems existing in the current AI course and put forward improvement suggestions (Huang, 2021). In the current social environment, with the introduction of relevant policies for MC to receive education in cities, a door is slowly opened for MC to receive urban education. To a certain extent, the relevant policies adopted in China are biased toward assimilation policies, such as urbanization and citizenization strategic development planning. The implementation effect is good, which is conducive to the educational integration of MC. The algorithm comparison results show that the results of ART-BP algorithm are 65 ∼ 96%, better than the results of similar algorithms. Therefore, the research results show that the use of AI technology combined with DL technology for modeling can establish an effective and accurate ART-BP evaluation model to effectively evaluate the impact of local education policies on the education of MC.

## Conclusion

This work studies and establishes the evaluation model of EP combining ART and DL technology for the educational integration of MC, thus analyzing the actual role of EP. First, it defines the migrant population and MC. Second, it introduces the principle of AI and DL technology. Furthermore, the data of education effect of MC are collected, and the ART-BP evaluation effect model is established. Finally, the evaluation model is used to test and analyze the effect of EP. The final experiment shows that the evaluation model of MC’s education integration based on ART-BP network can effectively and accurately evaluate the integration effect of MC’s education policies. Although this work basically achieved the original expected research objectives and obtained some valuable research conclusions, the research results still have certain limitations, and the research conclusions may be limited by the following factors. The data set processing needs to be further optimized, the different effects of policies in various places are not studied separately, and the reliability and rationality of the performance of the experimental platform and evaluation indicators are not verified. Therefore, the future research will further improve the model and further study the role of policies in different regions.

## Data Availability Statement

The raw data supporting the conclusions of this article will be made available by the authors, without undue reservation.

## Ethics Statement

The studies involving human participants were reviewed and approved by the Northeastern University Ethics Committee. The patients/participants provided their written informed consent to participate in this study. Written informed consent was obtained from the individual(s) for the publication of any potentially identifiable images or data included in this article.

## Author Contributions

All authors listed have made a substantial, direct, and intellectual contribution to the work, and approved it for publication.

## Conflict of Interest

The authors declare that the research was conducted in the absence of any commercial or financial relationships that could be construed as a potential conflict of interest.

## Publisher’s Note

All claims expressed in this article are solely those of the authors and do not necessarily represent those of their affiliated organizations, or those of the publisher, the editors and the reviewers. Any product that may be evaluated in this article, or claim that may be made by its manufacturer, is not guaranteed or endorsed by the publisher.
